# Navigating Challenges in Orbital Surgery: The Journey and Struggles in Iraq’s Neurosurgical Landscape

**DOI:** 10.7759/cureus.67914

**Published:** 2024-08-27

**Authors:** Ali Alshalchy, Sajjad G Al-Badri, Saleh A Saleh, Mohammed A Bani Saad, Mustafa Ismail

**Affiliations:** 1 Department of Neurosurgery, College of Medicine, University of Baghdad, Baghdad, IRQ; 2 Department of Surgery, College of Medicine, University of Baghdad, Baghdad, IRQ; 3 Department of Surgery, Baghdad Teaching Hospital, Medical City Complex, Baghdad, IRQ

**Keywords:** sociopolitical issues, resilience and improvement, facility inadequacy, training challenges, orbital surgery development

## Abstract

Orbital surgery in Iraq has undergone significant evolution, primarily influenced by periods of conflict that necessitated rapid advancements in surgical techniques. Despite pioneering efforts, such as the establishment of the first Orbital Surgery Center in Baghdad, the field grapples with multifaceted challenges. Training neurosurgeons remains a critical hurdle, hindered by inadequate programs, limited exposure to complex cases, and a scarcity of experienced mentors. These issues are compounded by insufficient medical facilities lacking essential equipment and resources vital for advanced procedures. Moreover, societal factors, notably tribalism, exert a profound impact on neurosurgical practice, often leading to disparities in resource allocation and posing threats to surgeons’ safety and professional integrity. This confluence of educational, infrastructural, and sociopolitical obstacles underscores the pressing need for comprehensive reforms to advance orbital surgery and ensure optimal patient care in Iraq.

## Editorial

Background

Orbital surgery, a specialized field within neurosurgery, plays a critical role in the management of complex conditions affecting the orbit, including tumors, trauma, congenital anomalies, and other pathologies. Despite its importance, the practice of orbital surgery in Iraq faces numerous challenges. This editorial delves into the historical development of orbital surgery in the country, tracing its evolution and the milestones that have shaped the field’s current state. A major focus of discussion is the training of neurosurgeons in Iraq which is hindered by several obstacles. These include inadequate access to specialized training programs, limited opportunities for hands-on experience, and a need for more advanced surgical facilities. These issues collectively contribute to a gap in the proficiency levels of surgeons, affecting the quality of care that patients receive [1,2].

Furthermore, the editorial sheds light on the broader neurosurgical practice in Iraq, where tribalism and other sociopolitical factors often influence the professional environment. Such dynamics can lead to disparities in access to training and resources and create barriers to collaboration and knowledge-sharing among surgeons. The lack of adequate facilities further compounds these challenges, making it difficult for surgeons to keep pace with advancements in the field and deliver optimal care.

History of orbital surgery in Iraq

Orbital surgery in Iraq has significantly evolved, particularly during and after times of conflict, where the necessity to address complex injuries became a driving force for innovation and skill development. The Iraq-Iran war, which lasted from 1980 to 1988, was marked by a high incidence of maxillofacial injuries due to the intense nature of the combat, including the widespread use of explosives and chemical weapons. These injuries often involved the orbital region, necessitating specialized surgical interventions. In response to the overwhelming number of severe facial injuries, Iraqi surgeons had to advance their skills and adopt new surgical techniques quickly. This period of conflict acted as a catalyst for the development of more sophisticated methods in orbital surgery. The urgency and volume of cases provided a unique, albeit challenging, environment for surgical advancements [1].

Notably, international collaboration played a crucial role in developing these surgical techniques. Surgeons such as Paul Tessier, a pioneer in craniofacial surgery, contributed significantly to this progress. Tessier and other international experts mentored local Iraqi surgeons, sharing their expertise and helping them master complex procedures. This mentorship not only improved the immediate care of war-related injuries but also laid the foundation for the continued development of orbital surgery in Iraq, allowing local surgeons to refine and innovate techniques that could be used in both military and civilian settings. The combination of necessity due to conflict and the influence of global surgical leaders has shaped the field of orbital surgery in Iraq, leading to advancements that have had a lasting impact on the region’s medical capabilities [1].

With his pioneering spirit, Prof. Abdul Hadi Al-Khalili has helped considerably establish and develop neurosurgery in Iraq. After graduating from Baghdad University in 1966 as a doctor of medicine and undergoing specialization in ophthalmology in the United Kingdom, Prof. Al-Khalili later changed the direction of his specialty to neurosurgery. He trained in Pinderfields Hospital in Yorkshire and General Infirmary in Leeds, becoming a fellow of the Royal College of Surgeons. Back in Iraq, he founded the first Orbital Surgery Center in Baghdad in 2002. This center is classified as a world standard by the World Health Organization and accredited by the American Academy of Ophthalmology. His work has laid the foundation for the practice of orbital surgery, but the specialty has continued to be tested by ongoing conflict and resource scarcity [2].

Challenges in training

One of the critical problems in advancing orbital surgery in Iraq is the need for well-established training programs in this field. The field of orbital surgery is one area that demands very high skill and a wide area of training, especially in microneurosurgery, as one performs inside a very limited area that contains high vascularity and nerve content. The training for microneurosurgery is highly demanding and requires double the time of other types of microsurgeries due to the intricacies involved. The deployment of surgical trainees in conflict zones often disrupts their formal education. As an example, the mobilization of surgical trainees during the Iraq conflict substantially upset the continuity of the training for them. Added to this is the need for more exposure to complex cases and structure in mentorship, contributing to a decline in the competence of general surgeons. Microneurosurgery training programs are one of the hardest due to the precision and complexity it involves. The skills in operation on extremely small spaces, highly vascular and full of nerves, have to be developed by the surgeon. This consumes much time but can only be effective with regular practice under guidance. One such drawback, for instance, in Iraq, is the need for more modern training facilities and experienced mentors in this field who would teach the aspirants the much-needed skills and experience. This gap in training is what reflects the poor advancement of orbital surgery in the country [3].

Challenges of inadequate facilities

This seriously impairs the quality of orbital surgery services in Iraq. Surgical teams based, for example, at Al Assad Airbase would likely have a low volume of surgical activities performed, with potentially long-term adverse effects on their skills in these procedures. This is enhanced by the shortfall in available surgical equipment and technologies required for performing complex orbital surgeries. This shows why there is a need for improved infrastructure and resources. The demands of conflict-related injuries are so high, but the level of capacity local medical facilities have is so low. In Iraq, the majority of hospitals and clinics need to be equipped and resourced to perform many, if not all, of the advanced surgical procedures. The deficit also extends to surgical tools, operating rooms, and advanced imaging equipment that form the basis for complex orbital procedures. In addition, even a few basic medical materials are scarcely available at any given time, thereby adding to the complication of medical situations and compelling surgeons to work under very poor conditions where they have to improvise. The need for more facilities, therefore, not only hampers instant surgical outcomes but also affects overall development and specialization in the field of orbital surgery [4].

Societal problems facing neurosurgery practice in Iraq

The practice of neurosurgery in Iraq is further tainted by the sociopolitical fabric of the country, with the paramount importance of the influence of tribalism. Tribal connections often determine the delivery of health services, as these tribal leaders could easily influence resources and development in local jurisdictions. Although tribalism has challenged and, at times, helped community-based healthcare initiatives since reemerging in post-2003 Iraq, it also tends to segregate. This can have dire consequences for surgeons as they stand at risk of being harmed physically and may lose their reputation. The consequences of risky surgeries in Iraq become more serious when a complication occurs - these procedures are already sensitive. They might demand financial compensation for the incident from a surgeon. In turn, under threat of violence, some may face insistent requests for this compensation to be paid to the families of their patients. This has been an incredibly stressful work environment, prompting many would-be surgeons to abandon the profession and some standing experts to leave the country. The lack of adequate legal frameworks to protect health professionals in Iraq is also a major risk for surgeons. Surgeons have repeatedly been subjected to trials by tribal structures that could fine them heavily, mete out social exile, and inflict other harsh penalties [5].

This situation led to constant change of hands among medical professionals, further pressing on the already limited healthcare resources. Violence can always break forth, and even the fear of such a system threatens to infringe upon the functionality of the surgeons, badly affecting their morale and confidence.

Conclusions

Addressing the challenges in orbital surgery in Iraq requires a multifaceted approach that includes enhancing training programs, improving medical facilities, and navigating the complex sociopolitical landscape. Prioritizing these areas will be crucial for advancing the field and ensuring that surgeons are well-equipped to provide high-quality care. A collaborative effort, both locally and internationally, is essential to overcome these obstacles and foster long-term improvements in neurosurgical practice in the country.

## References

[1] Hennocq Q, Bennedjaï A, Simon F (2019). Maxillofacial surgery in wartime Middle-East: Paul Tessier's missions to Iran. J Craniomaxillofac Surg.

[2] Al-Sharshahi ZF, Al-Hemiery HA, Albaqer HA, Hamandi YM, Al-Awadi OM, Hoz SS (2021). Following the footprints of pioneers on neurosurgery in Iraq: Abdul Hadi Al-Khalili. Surg Neurol Int.

[3] Krysa J (2004). Mobilised to Iraq: a surgical trainee's tale. BMJ.

[4] Vermeulen CF, Keijzers PJ, Fredriks EH, van der Hee P, Van Waes OJ, Hoencamp R (2019). Dutch combat operation experiences in Iraq and Afghanistan: the conundrum of low surgical workload deployments. Injury.

[5] Ismail M, Ayad F, Al-Ageely TA, Elamin O, Salih HR, Aljuboori Z, Hoz SS (2023). Societal challenges facing neurosurgeons in low- and middle-income countries: Iraq as an example. Surg Neurol Int.

